# Middle Ear Cavity and Mastoid Neuroendocrine Tumor Presenting as Otomastoiditis with Cholesteatoma: A Clinicoradiological and Histopathological Correlation

**DOI:** 10.1055/s-0043-1777695

**Published:** 2023-12-26

**Authors:** Ashwini Chalikandy, Sandip Basu

**Affiliations:** 1 Radiation Medicine Centre, Bhabha Atomic Research Centre, Tata Memorial Hospital Annexe, Parel, Mumbai, Maharashtra, India; 2 Homi Bhabha National Institute, Mumbai, Maharashtra, India

**Keywords:** neuroendocrine tumor, middle ear cavity, ^68^ Ga-DOTATATE-PET/CT, ^177^ Lu-DOTATATE PRRT

## Abstract

Neuroendocrine tumors of the middle ear are rare, comprising of less than 2% of primary tumors of the ear. The clinical and imaging findings of these tumors are nonspecific, and histological and immunohistochemical findings are confirmatory. Herein, we present a case of 48-year-old male, presenting with chief complaints of hearing loss of left ear with foul smelling discharge, with the initial clinical impression of otomastoiditis of the middle ear with cholesteatoma and being operated for the same, the final histopathology report inferred it as well-differentiated neuroendocrine tumor grade 1 with Ki-67 index less than 2%. Immunohistochemical examinations demonstrated positive staining of the tumor cells for cytokeratin, synaptophysin and chromogranin A, and negative for smooth muscle actin, desmin, S-100. The biochemical investigations showed raised serum chromogranin A levels. Based upon the findings on anatomical imaging modalities including high-resolution computed tomography temporal bone and magnetic resonance imaging paranasal sinuses (MRI PNS), the lesion was inferred inoperable due to involvement of dura of petrous apex, and therefore he was referred for consideration of peptide receptor radionuclide therapy (PRRT). MRI PNS also showed involvement of the horizontal part of facial nerve, indicating local aggressiveness of the tumor. ^68^ Ga-DOTATATE-PET/CT showed high-grade somatostatin receptor expressing soft tissue lesion involving middle ear and external auditory canal (Krenning's score 4), with low-grade metabolic activity on ^18^ F-FDG-PET/CT. The post-therapy scan following ^177^ Lu-DOTATATE PRRT, showed abnormal tracer concentration at the described site. Due to extreme rarity of this disease entity, it is important to accrue data for accurate diagnosis, proper management, and follow-up.

## Introduction

Neuroendocrine tumors (NETs) are widely heterogeneous group of tumors originating from diffuse neuroendocrine system. According to World Health Organization (WHO) 2010 classification system, the neuroendocrine neoplasms (NENs) are classified as NET grade-1(Ki- 67: < 3%), NET grade 2 (Ki-67: 3–20%), and poorly differentiated grade 3 NENs or neuroendocrine carcinoma (Ki-67: > 20%). The WHO 2017 classification recognized a subset termed as NET grade 3 with Ki-67 labeling index more than 20%. 1 They traditionally originate from gastrointestinal tract and lung and is rarely found in the middle ear. Other relatively rare reported primary sites include cervix, 2 uterine endometrium, 3 testis 4 and breast, 5 kidney, 6 urinary bladder, 7 and parotid gland. 8

NEN of the ear is very uncommon, accounting less than 2% of primary ear tumors. 9 The clinical course of these tumors is usually indolent. It tends to occur in fifth decade of life, but pediatric age group tumors are also reported. 10 11 These tumors have been termed differently in literature reports, such as carcinoid, middle ear adenoma, adenocarcinoma, adenomatous tumors, and adenocarcinoid. 9 10 It is postulated that NET of middle ear originates from an undifferentiated pluripotent endodermal stem cell, as epithelial cells with neuroendocrine features have not been identified in the middle ear. Even though these tumors are considered to be low aggressive neoplasm, long-term follow-up is needed in a series of patients. 10 The clinical and radiological findings are nonspecific for this tumor, and often cannot lead to definitive diagnosis. 12

As these tumors show somatostatin receptor (SSTR) expression, positron emission tomography/computed tomography (PET/CT) with ^68^ Ga-labeled somatostatin analogues (SSA) can be used for staging, along with theranostic pairing with ^177^ Lu-labeled agents, for metastatic/advanced neuroendocrine tumors (NETs). The treatment of choice is complete resection of tumor. Long-acting SSAs and peptide receptor radionuclide therapy (PRRT) have been used in well-differentiated metastatic NETs. Likewise, SSAs and PRRT can be considered in inoperable and/or metastatic NET of middle ear. 13 In this report, we present a rare case of inoperable NET of middle ear, who was considered and treated with PRRT.

## Case Report

A 48-year-old male, with no comorbidities presented with hearing loss of left ear associated with foul smelling discharge, initially diagnosed as otomastoiditis with cholesteatoma of left ear and was operated for the same. The histopathology report inferred as well-differentiated NET grade 1 with Ki-67 index less than 2%. Immunohistochemistry revealed tumor cell expression of cytokeratin, synaptophysin, and chromogranin A (CgA); and negative for SMA, desmin, S–100. On biochemical investigations, serum CgA level was found to be 120.9 µg/dL (normal: 7–25 µg/dL).

**Fig. 1:**
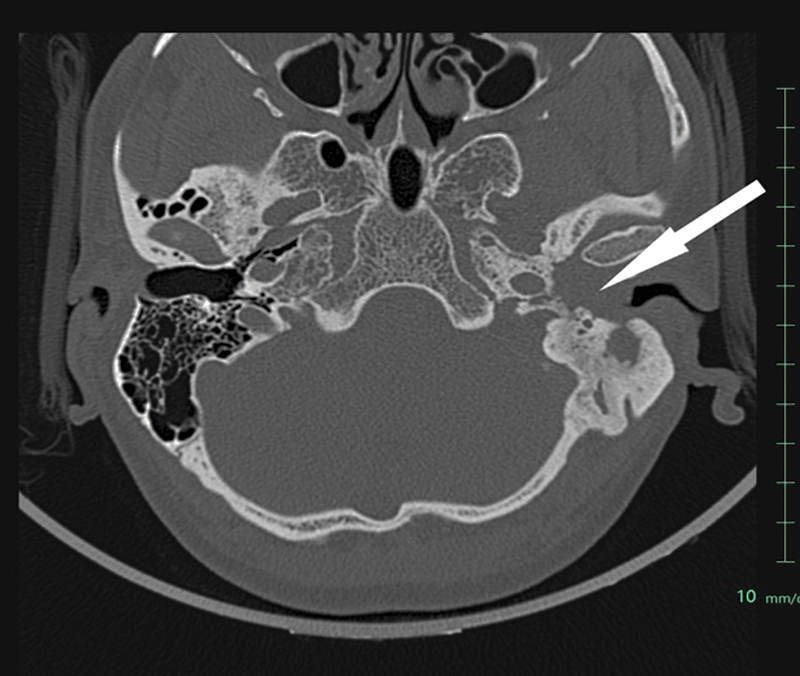
High-resolution computed tomography (HRCT) of temporal bone. HRCT of temporal bone ( *white arrow* ) demonstrating ill-defined soft tissue involving left external auditory canal and middle ear. There is extensive erosion of the squamous part of temporal bone with soft tissue extending into the middle ear and mastoid air cells. Mastoid air cells are completely sclerosed and facial nerve canal is completely eroded.

**Fig. 2:**
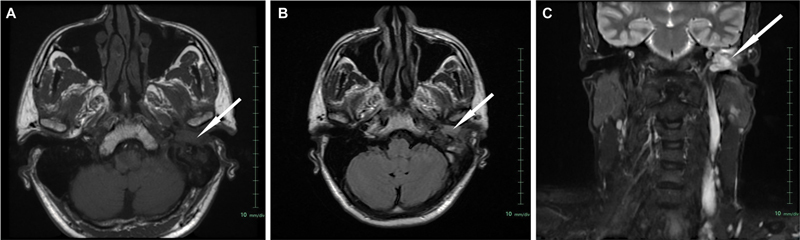
Magnetic resonance imaging showing ill-defined lobulated soft tissue lesion involving left external auditory canal and middle ear cavity and mastoid region measuring 3.1 × 2.4 × 2.5 cm (TR × AP × CC). The lesion is predominantly along the superior and posterior wall of the external auditory canal and erodes these walls. Medially it involves the middle ear cavity. There is extensive erosion of the squamous part of temporal bone with soft tissue extending into the middle ear and mastoid air cells. No obvious intracranial extension noted. Horizontal part of the facial nerve was involved. Soft tissue lesion ( *white arrow* ) was isointense on T1-weighted images and heterogenous on T2 fluid-attenuated inversion recovery and short tau inversion recovery (FLAIR and STIR) images. ( **A** ) T1-weighted, ( **B** ) T2 FLAIR, and ( **C** ) STIR.

^68^ Ga-DOTATATE-PET/CT ( Fig. 3 ) showed SSTR expression (maximum standardized uptake value [SUVmax]: 35.51) in the soft tissue lesion involving middle ear and left external auditory canal (Krenning score: 4). ^18^ F-FDG-PET/CT ( Figs. 4 , 5 ) showed low-grade metabolic activity (SUVmax: 6.93) of the corresponding lesion. As the lesion was considered to be inoperable (due to involvement of dura of the petrous apex) and showed high-grade SSTR expression with Krenning score 4, he was referred for the consideration of PRRT. He received 209 mCi ^177^ Lu-DOTATATE intravenously with standard regimen and the post-therapy scan showed abnormal tracer concentration at the level of middle ear and external auditory canal ( Fig. 6 ). He is being followed up at present for response.

High-resolution computed tomography of temporal bone showed an ill-defined soft tissue involving left external auditory canal and middle ear ( Fig. 1 ). There was extensive erosion of the squamous part of temporal bone with soft tissue extending into the middle ear and mastoid air cells. Mastoid air cells were completely sclerosed and facial nerve canal completely eroded. Magnetic resonance imaging paranasal sinuses (MRI PNS) and neck (temporal bone) showed ill-defined lobulated soft tissue lesion involving left external auditory canal and middle ear cavity and mastoid region measuring 3.1 × 2.4 × 2.5 cm (transverse [TR] × anteroposterior [AP] × craniocaudal [CC]). The lesion is predominantly along the superior and posterior wall of the external auditory canal and erodes these walls; medially it involves the middle ear cavity; there is extensive erosion of the squamous part of temporal bone with soft tissue extending into the middle ear and mastoid air cells. No obvious intracranial extension noted. Horizontal part of facial nerve is involved. It is isointense on T1-weighted images ( Fig. 2A ) and heterogeneous on T2 fluid-attenuated inversion recovery ( Fig. 2B ) and short tau inversion recovery ( Fig. 2C ) images.

**Fig. 3:**
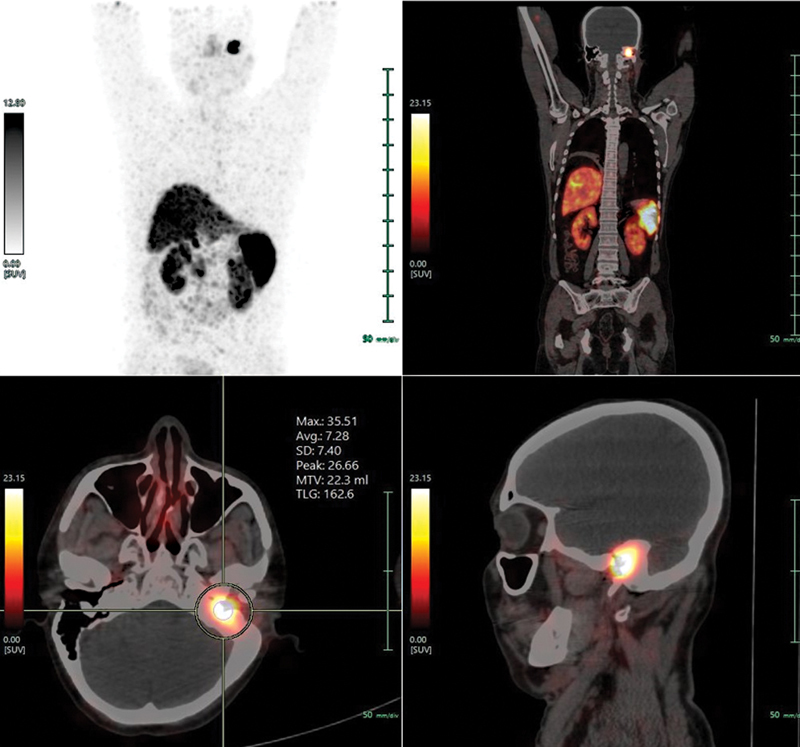
^68^ Ga-DOTATATE-PET/CT scan. ^68^ Ga-DOTATATE PET/CT showing somatostatin receptor expressing ill-defined soft tissue lesion (maximum standardized uptake value: 35.51) involving left external auditory canal and middle ear. Sclerosis of mastoid air cells noted.

**Fig. 4:**
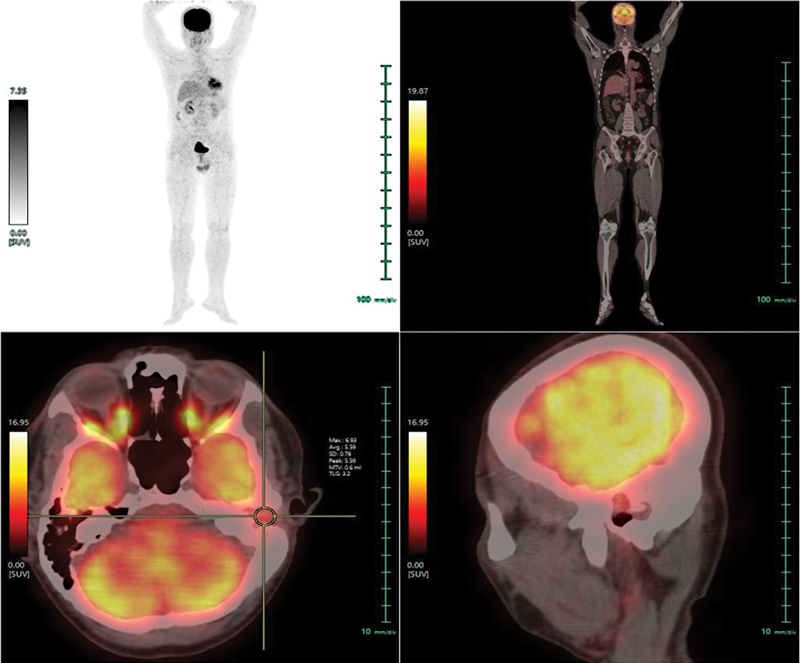
^18^ F FDG PET/CT scan. ^8^ F-FDG-PET/CT scan showing low-grade metabolic activity in the aforementioned ill-defined soft tissue lesion involving left external auditory canal and middle ear.

**Fig. 5:**
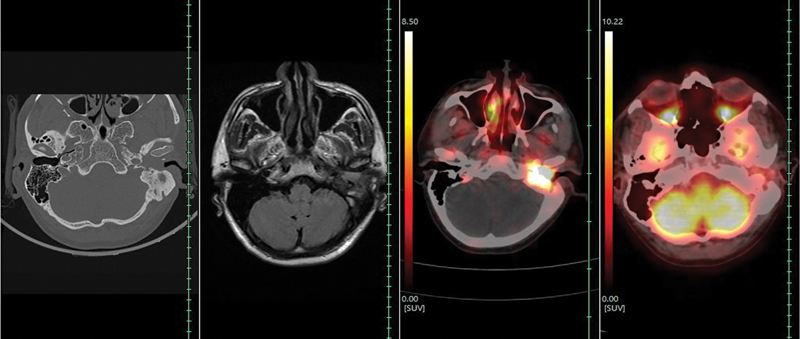
Comparison between contrast-enhanced computed tomography temporal bone, magnetic resonance imaging (paranasal sinuses and neck), ^68^ Ga-DOTATATE-PET/CT, and ^18^ F-FDG-PET/CT scan, at the lesion level.

**Fig 6:**
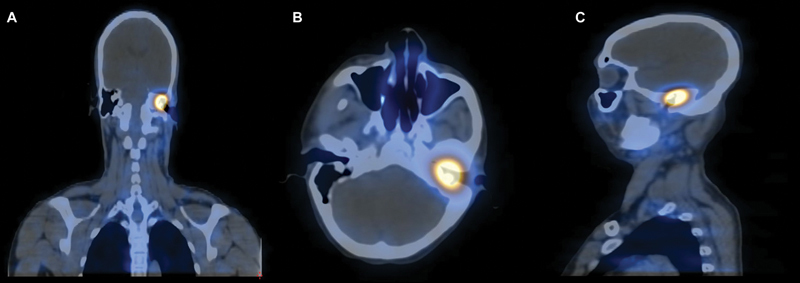
Post-therapy single-photon emission computed tomography/computed tomography (SPECT/CT) of ^177^ Lu-DOTATATE showing abnormal tracer concentration at the level of middle ear and external auditory canal. ( **A** ) Coronal SPECT/CT, ( **B** ) axial SPECT/CT, and ( **C** ) sagittal SPECT/CT.

## Discussion

Thus, in this report, we present on a rare case of inoperable, locally aggressive neuroendocrine rumor of middle ear and mastoid region, extending into external auditory canal, with the clinical, imaging features with histological correlation and management with PRRT. The usual presentations of these tumors are ear fullness, otalgia, hearing loss, and an intact eardrum behind a visible brown-red mass. As in our case, cholesteatoma is the usually first suspect. Other differential diagnosis includes jugulotympanic paraganglioma, meningiomas, acoustic neuroma, adenocarcinoma, and rhabdomyosarcoma. It is important to differentiate the different middle ear tumors, because the managements are significantly different. Being a rare tumor, the diagnosis of NET is often delayed. However, histological, imaging, and immunohistochemical features often lead to accurate diagnosis. 14

Asa et al showed that these tumors are of low proliferative grade but can occasionally have high Ki-67 indices. Similar to other NETs, these tumors show a spectrum of behavior, and can be graded as G1, G2, and G3 NET. 15 Several authors have proposed different classification of these tumors, because it tends to exhibit local invasion, local recurrence, and rarely shows metastatic potential. Saliba and Evrard classified middle ear glandular neoplasm into three types; type 1 is the most common type, which is neuroendocrine adenoma of middle ear (positive immunohistochemistry and negative metastasis), type II is middle ear adenoma (negative immunochemistry and negative metastasis), and the least common type III is carcinoid tumor of the middle ear (positive immunohistochemistry, positive metastasis and/or carcinoid syndrome). 16 Marinelli et al conducted a mutli-institutional retrospective study of 32 cases and proposed “T/N/M/S” staging system. 17

There is relative paucity of published literature on middle ear NET. Conventional CT and MRI scans can provide details regarding anatomical staging but is unable to identify specific tumor types. Therefore, usually middle ear NET remains a histopathological diagnosis. SSA PET/CT can be used functional evaluation of these tumors. The overall sensitivity of ^68^ Ga-DOTATATE PET/ CT in NET is of 92%, which includes middle ear NET, although it is not entirely specific. 18 Even though ^68^ Ga-DOTATATE PET/CT is rarely utilized preoperatively in the presence of middle ear mass, it is used mainly in postoperative assessment in patients with residual or metastatic tumor. It also plays a crucial role in determining possible treatment options for well-differentiated NETs. Thus, as in our case, which was inoperable due to involvement of dura of petrous apex, it can substantiate the role of ^68^ Ga-DOTATATE PET/CT scan for SSA therapy as well as PRRT.

van der Lans et al conducted a retrospective case review of nine patients diagnosed with middle ear adenomatous neuroendocrine tumors (MEANTs). One patient had locally invasive tumor like the presently described case. Other patients had locally nonaggressive tumor confined to tympano-mastoid space. Among these, five patients presented with recurrence, even after successful surgery. This study reported an unpredictable tumor behavior, a high tendency for recurrence, and lack of predictive histopathological/immunohistochemical markers in MEANTs. 19

While initial reports on middle ear carcinoids have previously considered this as benign entity with no metastasis, a critical review of the literature demonstrates a small fraction of cases, in which middle ear carcinoid tumors have given rise to regional (cervical node) and distant (bone, liver) metastases. Yang et al reported a case of MEANT with ipsilateral local brain, bone, nerve invasion, and bilateral cervical lymphadenopathy. 20 Likewise, Mooney et al, 21 Menezes and Wakely et al, 22 and Pellini et al 23 reported isolated cases of middle ear carcinoid tumor with recurrences and locoregional metastases. Fundakowski et al demonstrated middle ear carcinoid with distant osseous metastasis. 24 Another case reported by Gaafar et al showed middle ear carcinoid with liver metastasis. 25

In the present case, the tumor involved middle ear and external auditory canal, with extensive erosion of squamous part of temporal bone and involvement of horizontal part of facial nerve. The possibility of tumor spreading from another neuroendocrine site and presence of distant metastasis was excluded by whole body PET scan (a dual tracer PET/ CT with ^68^ Ga-DOTATATE and ^18^ F-FDG PET/ CT was used for baseline evaluation). The patient will be followed up clinically (symptomatic evaluation and otoscopic examination) and by radiological imaging (MRI and SSTR-based PET/CT) to assess the response to administered PRRT and to accue further data on this rare malignancy.
